# The burden of common variable immunodeficiency disorders: a retrospective analysis of the European Society for Immunodeficiency (ESID) registry data

**DOI:** 10.1186/s13023-018-0941-0

**Published:** 2018-11-12

**Authors:** Irina Odnoletkova, Gerhard Kindle, Isabella Quinti, Bodo Grimbacher, Viviane Knerr, Benjamin Gathmann, Stephan Ehl, Nizar Mahlaoui, Philippe Van Wilder, Kris Bogaerts, Esther de Vries, in collaboration with the Plasma Protein Therapeutics Association (PPTA) Taskforce, Albert Farrugia, Shanthy Krishnarajah, Joan Mendivil, Mercedes Prior, Tim Rübesam, Michael Runken

**Affiliations:** 1 Plasma Protein Therapeutics Association, Boulevard Brand Whitlock 114b4, 1200 Brussels, Belgium; 2 Faculty of Medicine and Health Sciences, Ghent University, C. Heymanslaan 10, 9000 Ghent, Belgium; 3 The ESID Registry Working Party, https://esid.org/Working-Parties/Registry; 4 Center for Chronic Immunodeficiency, Medical Center – University of Freiburg, Faculty of Medicine, University of Freiburg, Freiburg, Germany; 5 Department of Molecular Medicine, Sapienza University of Rome, Rome, Italy; 6 University Hospital Policlinico Umberto I, Rome, Italy; 7 Institute of Immunology and Transplantation, Royal Free Hospital, University College London, London, UK; 8 French National Reference Center for Primary Immune Deficiencies (CEREDIH) and Pediatric Immuno-Haematology and Rheumatology Unit Necker-Enfants Malades University Hospital, Assistance Publique-Hôpitaux de Paris, Paris, France; 9 Paris Descartes University, Sorbonne Paris Cité, Imagine Institute, Paris, France; 10 INSERM UMR 1163, Laboratory of Human Genetics of Infectious Diseases, Necker Branch, Paris, France; 11 Centre de recherche en Economie de la Santé, Gestion des Institutions de Soins et Sciences Infirmières, Ecole de Santé Publique, University of Brussels (ULB), Brussels, Belgium; 12 Interuniversity Institute for Biostatistics and Statistical Bioinformatics (I-BioStat), KU Leuven – University of Leuven, I-BioStat, 3000 Leuven, Belgium; 13 University Hasselt, I-BioStat, 3500 Hasselt, Belgium; 14 Department Tranzo, Tilburg University, PO Box 90153 (RP219), 5000 LE Tilburg, the Netherlands; 15 Laboratory for Microbiology and Immunology, Elisabeth Tweesteden Hospital, PO Box 90151 (route 90), 5000LC Tilburg, the Netherlands

**Keywords:** Primary immunodeficiency, Primary antibody deficiency, Common variable immunodeficiency, Burden of disease, DALY, Health economics, Diagnostic delay

## Abstract

**Background:**

Common variable immunodeficiency disorders (CVID) are a group of rare innate disorders characterized by specific antibody deficiency and increased rates of infections, comorbidities and mortality. The burden of CVID in Europe has not been previously estimated. We performed a retrospective analysis of the European Society for Immunodeficiencies (ESID) registry data on the subset of patients classified by their immunologist as CVID and treated between 2004 and 2014. The registered deaths and comorbidities were used to calculate the annual average age-standardized rates of Years of Life Lost to premature death (YLL), Years Lost to Disability (YLD) and Disability Adjusted Life Years (DALY=YLL + YLD). These outcomes were expressed as a rate per 10^5^ of the CVID cohort (the individual disease burden), and of the general population (the societal disease burden).

**Results:**

Data of 2700 patients from 23 countries were analysed. Annual comorbidity rates: bronchiectasis, 21.9%; autoimmunity, 23.2%; digestive disorders, 15.6%; solid cancers, 5.5%; lymphoma, 3.8%, exceeded the prevalence in the general population by a factor of 34.0, 7.6, 8.1, 2.4 and 32.6, respectively. The comorbidities of CVID caused 8722 (6069; 12,363) YLD/10^5^ in this cohort, whereas 44% of disability burden was attributable to infections and bronchiectasis. The total individual burden of CVID was 36,785 (33,078, 41,380) DALY/10^5^. With estimated CVID prevalence of ~ 1/ 25,000, the societal burden of CVID ensued 1.5 (1.3, 1.7) DALY/10^5^ of the general population.

In exploratory analysis, increased mortality was associated with solid tumor, HR (95% CI): 2.69 (1.10; 6.57) *p* = 0.030, lymphoma: 5.48 (2.36; 12.71) *p* < .0001 and granulomatous-lymphocytic interstitial lung disease: 4.85 (1.63; 14.39) *p* = 0.005. Diagnostic delay (median: 4 years) was associated with a higher risk of death: 1.04 (1.02; 1.06) *p* = .0003, bronchiectasis: 1.03 (1.01; 1.04) *p* = .0001, solid tumor: 1.08 (1.04; 1.11) p < .0001 and enteropathy: 1.02 (1.00; 1.05) *p* = .0447 and stayed unchanged over four decades (*p* = .228).

**Conclusions:**

While the *societal* burden of CVID may seem moderate, it is severe to the *individual patient*. Delay in CVID diagnosis may constitute a modifiable risk factor of serious comorbidities and death but showed no improvement. Tools supporting timely CVID diagnosis should be developed with high priority.

**Electronic supplementary material:**

The online version of this article (10.1186/s13023-018-0941-0) contains supplementary material, which is available to authorized users.

## Introduction

Common variable immunodeficiency disorders (CVID) constitute a heterogeneous immune defect characterized by hypogammaglobulinemia, failure of specific antibody production, susceptibility to infections, and an array of comorbidities [1, 2]. CVID is one of the most prevalent types of primary immunodeficiencies, occurring in about 1: 25,000 of the population, equally affecting men and women [3–6]. CVID is typically characterized by significantly decreased levels of IgG, in combination with decreased IgA and/or IgM, poor vaccine response, and increased susceptibility to bacterial infections [3, 7]. A peak in the onset of symptoms falls in the first and third decades of life [3]. CVID can occur at any age but should not be diagnosed before the age of four, because other primary immunodeficiencies or transient hypogammaglobulinemia of infancy are at first difficult to distinguish and more likely in young infants [3, 7]. Although a full unanimity regarding the definition of CVID does not exist at this point, a recent International Consensus on Common Variable Immunodeficiency Disorders (ICON) offers a good framework for the diagnosis of CVID [3].

CVID is associated with high comorbidity and increased mortality [2–4, 8–13]. The most prominent clinical problems in CVID observed at diagnosis and during follow-up are recurrent respiratory tract infections, such as chronic sinusitis, chronic otitis media, bronchitis and pneumonia [7, 14, 15].

Complications of CVID can be divided into structural damage due to severe and/or recurrent infections such as bronchiectasis, and the consequences of the immune dysregulation [2, 10]. The latter ‘non-infectious’ complications of CVID are autoimmune and autoinflammatory conditions, such as cytopenias, granulomas, gastrointestinal inflammatory disease, enteropathy and splenomegaly [3, 9]. CVID is also associated with a higher prevalence of solid tumours and lymphoid malignancies [9]. In the past 40 years, the standard treatment of CVID has been immunoglobulin replacement therapy. It commenced with intramuscular products, which were abandoned once safe intravenous (IVIG) and subcutaneous (SCIG) therapies were introduced [9]. Recent research advocates the individualization of the immunoglobulin dose, depending not so much on trough IgG levels but on the incidence of infections [15–17].

The survival of people with CVID improved from about 30% 12 years after diagnosis reported in the first studied UK cohort in 1969 [18], to 58% 45 years after diagnosis as shown in a recent analysis [2]. Such improvements are believed to be associated with a better understanding of the disease, widespread usage of IgG replacement therapy and improved anti-microbial therapies, together resulting in a reduced incidence of severe infections [3, 10, 15–17, 19]. However, morbidity and mortality remain grave concerns for CVID patients [2, 3, 13]. The most common causes of death in CVID are reported to be respiratory failure from chronic lung disease, lymphomas and other cancers [3, 20]. Overall survival of people with CVID continues to be less than that of age-matched controls [3, 12, 20].

The burden of CVID in Europe in terms of loss of healthy life years due to premature death and disability has not been previously estimated. Burden of disease studies provide the initial indication of how the systems of care affect patient outcomes. The methodology for such burden of disease analysis was developed by the World Health Organization (WHO) and applied in a range of studies published as “Global Burden of Disease Study” (“GBD”) [21]. GBD uses Disability Adjusted Life Years (DALYs) as a common metric for the quantification of health loss, calculated as a sum of life years lost to premature mortality and life years lost to disability. DALYs allow for direct comparison of burden across diseases and geographic areas. Regular re-assessment of the burden of disease is crucial to track the evolution in clinical outcomes, to assess treatment and/or prevention campaigns results, and to define health service and research priorities. Moreover, results of burden of disease studies provide input for health economic evaluations of healthcare interventions.

GBD studies already analyzed the burden of more than 300 conditions across the globe, however, the burden of many rare diseases remains unknown. The objective of this research was to estimate the burden of CVID by using the data of the European Society for Immunodeficiencies (ESID) registry, the largest primary immunodeficiency registry in the world [22]. Despite certain limitations common to registry data in general, such as incomplete documentation and quality control [11], the ESID registry provides a valuable source of information for a burden of CVID analysis, due to the large size of the cohort.

## Methods

### Design

Retrospective analysis of the ESID registry data subset of patients classified as CVID by an immunologist and treated between 2004 and 2014.

### ESID registry

ESID registry is an electronic database for a uniform collection of demographic, clinical and immunological data on patients with primary immunodeficiency, established in 2004. The immunological treatment centres from most European countries contributed patient data to this database. The data from the patients’ clinical files were entered in the registry manually by treatment centre assistants. The registry is technically maintained at the Centre for Chronic Immunodeficiency, University Medical Centre Freiburg, Germany. The included patients signed a consent form (https://esid.org/Working-Parties/Registry/Informed-Patient-Consent). The data extraction was performed by the registry custodian (GK) based on CVID classification established by the immunological treatment centre and upon approval of the study design by the ESID Registry Steering Committee (Additional file 1: Overview of the ESID registry data used in this study).

### Patient inclusion

Patients were included in the analysis if within the ESID registry, they were classified as CVID by their treating immunologist^1^; with diagnosis of CVID established or confirmed after 4 years of age; and if they were treated in a centre between 2004 and 2014; and at least the following data were available: sex, country of origin, year of birth, year of CVID diagnosis, follow-up period. Patients with ‘old’ records, i.e. preceding the year of the ESID registry setup (2004) were not included.

### Data quality assessment

To exclude unreliable data from the analysis, the data found in the registry were examined for consistency with the coding rules. For numerical data, such as year of birth, visit date and Ig dose, a plausible range was established a priori; for the weight outcomes in children, the WHO child growth statistics were used [23]. Consistency of the ICD-10 codes and the textual descriptions of comorbidities and infections was checked. Unreliable or inconsistent data were removed and analysed as ‘missing’.

### Outcomes

Mortality, Years of Life Lost to premature death (YLL), prevalence of comorbidities, Years Lost due to Disability (YLD) and Disability Adjusted Life Years (DALY) in the ESID cohort were analysed over the period 2004–2014 and compared with the respective outcomes in the general population in Europe. Mortality rate was defined as average annual all-cause mortality rate. YLL were computed by multiplying the number of deaths in each age subgroup by the standard life expectancy at that age. The division in age subgroups was based on a 5-year age interval; age, sex, and country specific healthy life expectancy statistics were used [24].

YLD were estimated based on the GBD methodology: within the GBD studies, disability weights for above 300 conditions were estimated and assigned an index between 0 and 1, wherein 1 is associated with death and 0 with perfect health; annual YLD rate was then calculated as the prevalence of a condition in a particular year multiplied by the respective disability weight [25]. YLD associated with CVID was computed as a sum of YLDs caused by CVID comorbidities. YLD due to each comorbidity identified in the CVID cohort was calculated as follows: annual average YLD rate over the study period (2004–2014) in the general population as reported by the GBD study, divided by the annual average prevalence rate of the respective comorbidity in the general population over the same period, and multiplied by the annual average registration rate in the CVID cohort.

The registration rate of non-infectious and infectious comorbidities was derived from the respective subsets of patients with registered comorbidities by using the total number of patients in these subsets as denominator [11]. Non-infectious comorbidities were grouped as follows: bronchiectasis; granulomatous-lymphocytic interstitial lung disease (GLILD); splenomegaly; autoimmunity (cytopenias; and organ/systemic); granuloma (other than GLILD); enteropathy; solid tumor; lymphoma; lymphoproliferation; other chronic lung disease (asthma, COPD, emphysema) [2, 3, 9–11]. Infections were grouped in serious bacterial infections, such as pneumonia and meningitis [26]; and other infections, according to the GBD classification: lower respiratory (e.g. bronchitis); upper respiratory (e.g. sinusitis); otitis media; diarrhea; varicella/herpes zoster; other [24, 27].

Total *individual* disease burden was defined as annual average age-standardized DALY calculated as the sum of YLL and YLD rates per 10^5^ in this cohort. The *societal* disease burden, i.e. health loss caused by CVID per 10^5^ of general population, was calculated as DALY rate in the CVID cohort multiplied by the estimated CVID prevalence in Europe. The individual and societal burden of CVID and ten major causes of health loss in Europe were compared [24].

### Statistical analysis

The analyses were performed with SAS, version 9.4. Baseline characteristics were summarized by mean, standard deviation, median and range for continuous variables, and by numbers and percentages for categorical variables. The following formulas were applied: a) Annual death rate per 10^5^ = (N of deaths in year X) / (N of people in the cohort in year X) × 10^5^; b) Age-specific death rate = (N of deaths in year X in age group Y) / (N of people in the cohort in year X in age group Y) × 10^5^; c) Age-adjusted death rate = ∑((N of deaths in year X in age group Y) / (N of people in the cohort in year X in age group Y) × 10^5^ x Proportion of age group Y in world population).

The period prevalence of comorbidities was computed as number of cases with a comorbidity registered at least once during the follow-up period divided by the number of all patients in the subset with registered comorbidities. The annual prevalence over the period of 2004–2014 was computed by using a multiple imputation methodology for missing years of the diagnosis of the registered comorbidities and infections. Ten imputations for the missing year of a comorbidity or infection were drawn from a uniform distribution between the year of diagnosis and the last year of follow-up. If the duration of the infection was missing, it was sampled from a Poisson distribution mimicking the distribution of the durations of the observed infections. Age-standardization was performed by using the WHO world population standard [21].

All-cause mortality since time point of diagnosis was estimated using a Cox proportional hazards model with Efron’s method of tie handling and accommodating for left truncation (entry from 2004). The follow-up period was computed as the year of the last record minus the year of the CVID diagnosis. Association between survival and the following variables was explored: sex, age at diagnosis, age at onset, diagnostic delay, parental consanguinity, monthly Ig replacement dosage, prevalence of comorbidities. These associations were tested by means of univariable analysis and as bivariable analysis after adjustment for the age of CVID and the age of CVID symptoms onset, respectively. Diagnostic delay was explored as factor of the prevalence of comorbidities. Results were summarized by means of the hazard ratio (HR) and a 95% confidence interval (CI). Comorbidities and monthly Ig replacement dosage were handled as a time-dependent covariate. For the calculation of the mean monthly relative Ig dose, all registered dosages were converted in mg/kg. If only absolute Ig dose was available, the registered weight was used to calculate the relative monthly dose.

## Results

### Patient inclusion and characteristics

**Fig. 1:**
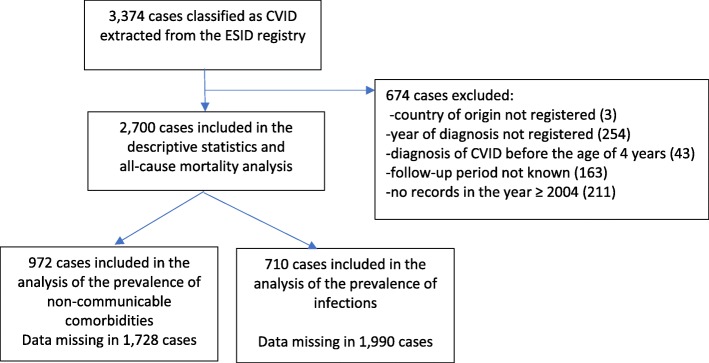
Inclusion of cases in the Burden of CVID analysis

From 3374 cases originally extracted from the ESID registry based on the recorded CVID diagnosis, 2700 were included in the analysis (Fig. 1). In total, 674 cases were excluded whereof 420 due to missing data on the country of residence (*n* = 3), year of CVID diagnosis (*n* = 254), follow-up period (*n* = 163); 211 patients had no records between 2004 and 2014; 43 patients were diagnosed before the age of 4 years without any records at an older age.

**Fig. 2:**
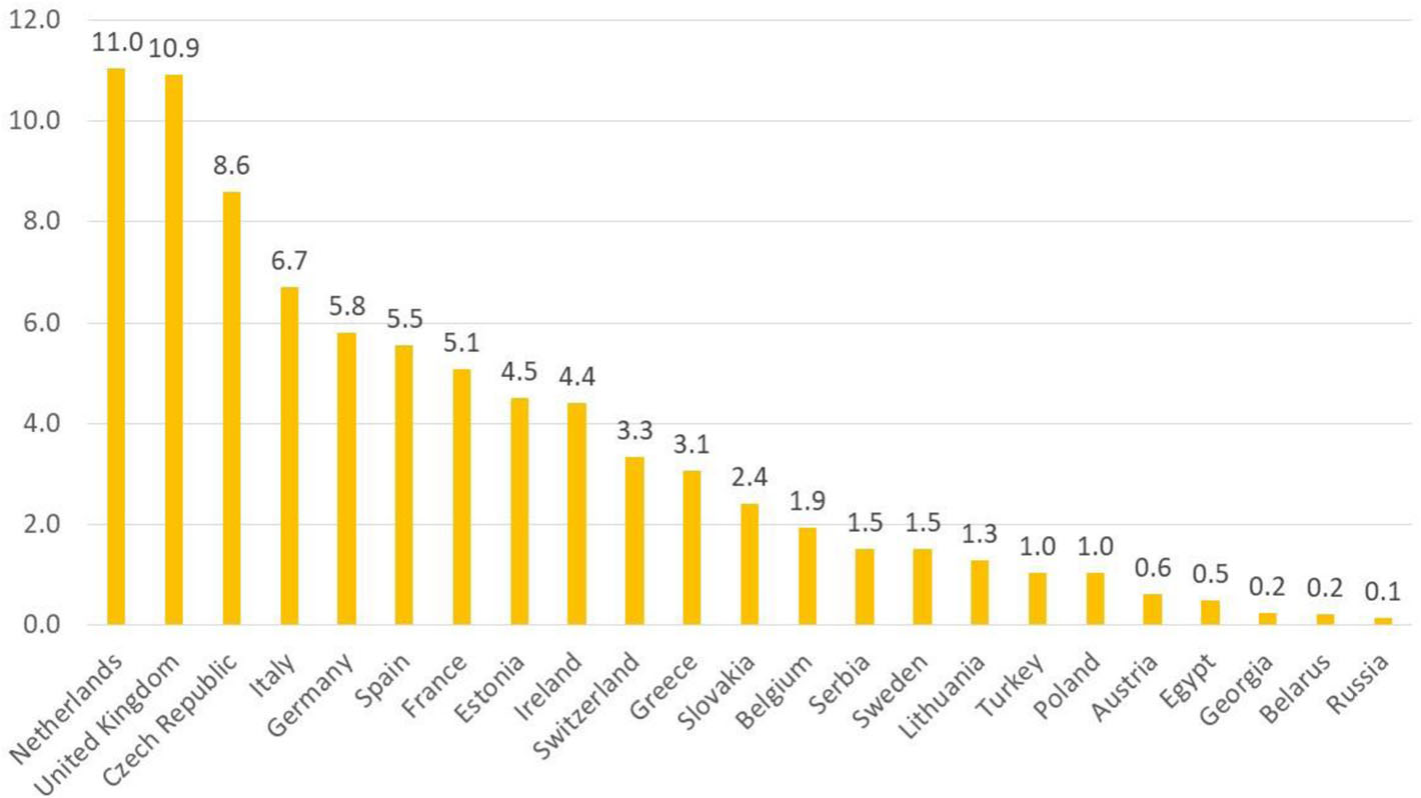
Registration rate of CVID patients in the ESID registry, per 1 million by country population

The included patients originated from 23 countries, whereof 2435 (90.2%) from Western Europe. The registration rate per million of country population varied between 0.1 (Russia) and 11.0 (Netherlands) (Fig. 2). Overall, 30.5% were diagnosed before the age of 18 years. There was a great variability in the proportion of pediatric patients per country: from none (Lithuania) to 100% (Poland, Russia, Belarus, Egypt, Georgia). The total follow-up period was 24,366 person-years with a per-patient median of 6 years (Table 1).

**Table 1:** The number of patients, registration rate, percentage of pediatric patients, follow-up period and mean monthly Ig dose per country

Country	Number of patients in the registry	Rate per 1 million of population	Percentage of patients diagnosed before 18 y.o.	Median (mean) follow-up period, years	Total person years	Percentage of patients with identified relative monthly Ig dose	Mean (SD) monthly Ig dose, mg/kg
Austria	5	0.6	80.0%	9.0 (10.6)	53	80%	450 (327)
Belarus	2	0.2	100%	3.0 (3.0)	6	N.A.	N.A.
Belgium	21	1.9	71.4%	6.0 (7.7)	162	61.9%	431 (110)
Czech Republic	90	8.6	30.0%	11.0 (12.1)	1093	80%	266 (146)
Egypt	4	0.5	100%	12.0 (10.3)	41	75%	300 (265)
Estonia	6	4.5	16.7%	6.5 (5.5)	33	100%	361 (70)
France	319	5.1	18.8%	6.0 (10.5)	3334	65.8%	538 (178)
Georgia	1	0.2	100%	5.0 (5.0)	5	100%	369 (−)
Germany	475	5.8	34.5%	7.0 (9.2)	4377	71.6%	385 (184)
Greece	34	3.1	76.5%	6.0 (9.3)	317	67.6%	544 (182)
Ireland	20	4.4	15.0%	15.5 (15.1)	302	50%	510 (68)
Italy	397	6.7	27.7%	7.0 (8.7)	3470	56.4%	405 (218)
Lithuania	4	1.3	0.0%	4.0 (6.5)	26	75%	380 (99)
Netherlands	183	11.0	38.3%	8.0 (9.9)	1803	56.3%	508 (214)
Poland	39	1.0	100%	5.0 (5.6)	218	100%	430 (142)
Russia	20	0.1	100.0%	6.0 (5.5)	110	90%	442 (146)
Serbia	11	1.5	81.8%	4.0 (5.5)	60	90.9%	400 (0)
Slovakia	13	2.4	84.6%	3.0 (5.5)	71	100%	392 (156)
Spain	258	5.5	24.4%	1.0 (3.4)	884	12.8%	412 (175)
Sweden	14	1.5	7.1%	8.0 (10.0)	140	78.6%	498 (221)
Switzerland	26	3.3	30.8%	7.0 (7.6)	197	69.2%	477 (287)
Turkey	75	1.0	76.0%	5.0 (5.6)	420	72%	472 (107)
U.K.	683	10.9	18.9%	7.0 (10.6)	7244	52.6%	528 (165)
Total/ overall	2700	3.9	30.5%	6.0 (9.0)	24,366	58.0%	454 (196)

*Ig* immunoglobulin, *N.A.* data not available, *y.o.* years old

The year of CVID diagnosis was ≤1980 in 3.7%; between 1981 and 1999 in 27.2%; and ≥ 2000 in 69.1% of patients. The median (min; max) age at diagnosis was 31 (4; 89) years, 26 (4; 83) in males versus 34 (4; 89) in females (independent samples Mann-Whitney U test, *p* < .001*)*. The overall proportion of male patients was 47.9% but higher in children and lower in adults: 56.9% among those diagnosed before the age of 18 years, and 43.0% among those diagnosed as adults.

The median (min; max) age at onset of symptoms was 18 (0; 81). The onset of CVID occurred at all ages, with the largest proportion (37.1%) between 0 and 11 years. The median (min; max) diagnostic delay was 4 years (0; 69). The diagnosis of CVID was established in the year of disease onset in 16.0% of the patients (*n* = 357) (Table 2).

**Table 2:** Patient characteristics

Characteristics	Data completeness, n (%)	
Sex, n (%)	2700 (100%)	
Male		1294 (47.9)
Female		1406 (52.1)
Age at diagnosis, years,	2700 (100%)	
Median (Min; Max)		31.0 (4; 89)
Mean (SD)		31.4 (19.6)
N (%)		
4–10		460 (17.0)
11–20		475 (17.6)
21–40		939 (34.8)
41–60		565 (21.0)
> 60		261 (9.7)
Age at onset, years,	2236 (82.8%)	
Median (Min; Max)		18.0 (0; 81)
Mean (SD)		22.4 (19.0)
N (%)		
≤ 10		829 (37.1)
11–20		401 (17.9)
21–40		601 (26.9)
41–60		309 (13.8)
> 60		96 (4.3)
Diagnostic delay, years	2236 (82.8%)	
Median (Min; Max)		4.0 (0; 69)
Mean (SD)		8.8 (11.4)
N (%)		
0 (diagnosis in the year of symptoms onset)		357 (16.0)
1–4		769 (34.4)
5–9		443 (19.8)
10–20		380 (17.0)
≥ 21		287 (12.8)
Period of diagnosis, n (%)	2700 (100%)	
≤ 1980		101 (3.7)
1980–1999		734 (27.2)
≥ 2000		1865 (69.1)
Consanguinity, n (%)	1488 (55.1%)	
With consanguinity		68 (4.6)
With records on immunoglobulin replacement therapy, n (%)	2290 (84.8%)	

Data on parental consanguinity indicating whether the parents or other ancestors (e.g. grandparents) of the patient are genetically related, were registered in 55.1% of patients. Of these, 4.6% (*n* = 68) were reported as offspring of consanguineous parents (Table 2).

The Ig replacement therapy was registered in 84.8% of the patients, with most of the dose records (82.4%) listed as absolute dose. Body weight was available in 52.5% of cases. After removing erratic records of the Ig dose (3.6%) and weight (2.3%), the relative monthly Ig dose could be analyzed in 1567 (58.0%) patients. The mean (SD) relative monthly dose was 454 (196) mg/kg, with a significant difference between countries *p* < .0001; the mean dose was lowest in the Czech Republic (266 mg/kg) and highest in Greece (544 mg/kg) (Table 1).

### Mortality and years of life lost to premature death

**Fig. 3:**
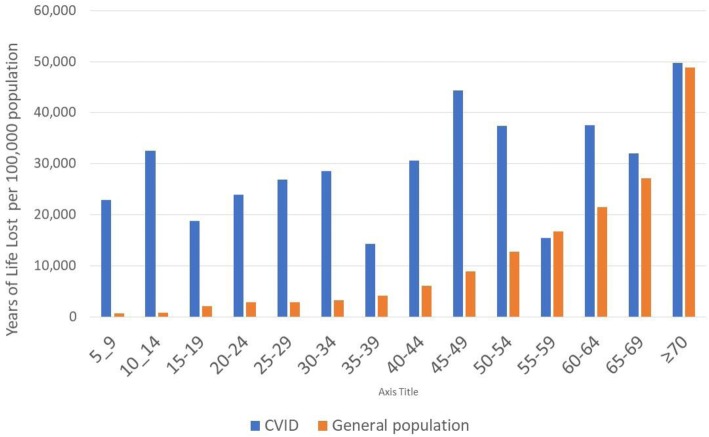
Annual average rate of Years of Life Lost to premature death, per 5-year age interval, over the period: 2004–2014. CVID cohort versus general population*. All causes, both sexes. *Source: Global Burden of Disease Studies, Western Europe: http://ghdx.healthdata.org/gbd-results-tool

The all-cause mortality was analysed from the records of all included patients (*n* = 2700). Death was registered in 102 patients (3.8%), aged between 6 and 84 years. This corresponded with 3372 Years of Life Lost due to premature death (YLLs). The annual average standardized rates per 10^5^ (95% CI) were 865 (678; 1052) deaths and 28,013 (27,009; 29,017) YLLs, exceeding the respective rates in the general population by a factor of 1.7 and 3.0. The death rates were higher than in the general population: in children aged 5 to 14, by a factor of 38; in patients between 15 and 34 years of age, by a factor of 8.5 to 9; in those aged 35 to 54, by a factor of 3.0 to 5.3; in patients aged 55 or older, by a factor of 0.6 to 1.9 (Fig. 3 and Additional file 2).

### Comorbidities of CVID

Concomitant diseases and infections were registered in 972 (36.0%) and 710 (26.3%) patients, respectively. There was a high consistency in the ICD-10 codes and the textual diagnostic descriptions (99.9%). The crude period prevalence rates of CVID comorbidities were largely consistent with the previously reported findings: bronchiectasis, 26.8%; splenomegaly, 24.0%; autoimmunity, 25.5%; neoplasms, 14.1%; enteropathy, 9.9%; granuloma, 9.1% (Additional file 3).

### Annual age-standardized prevalence of comorbidities and years of life lost to disability

**Fig. 4:**
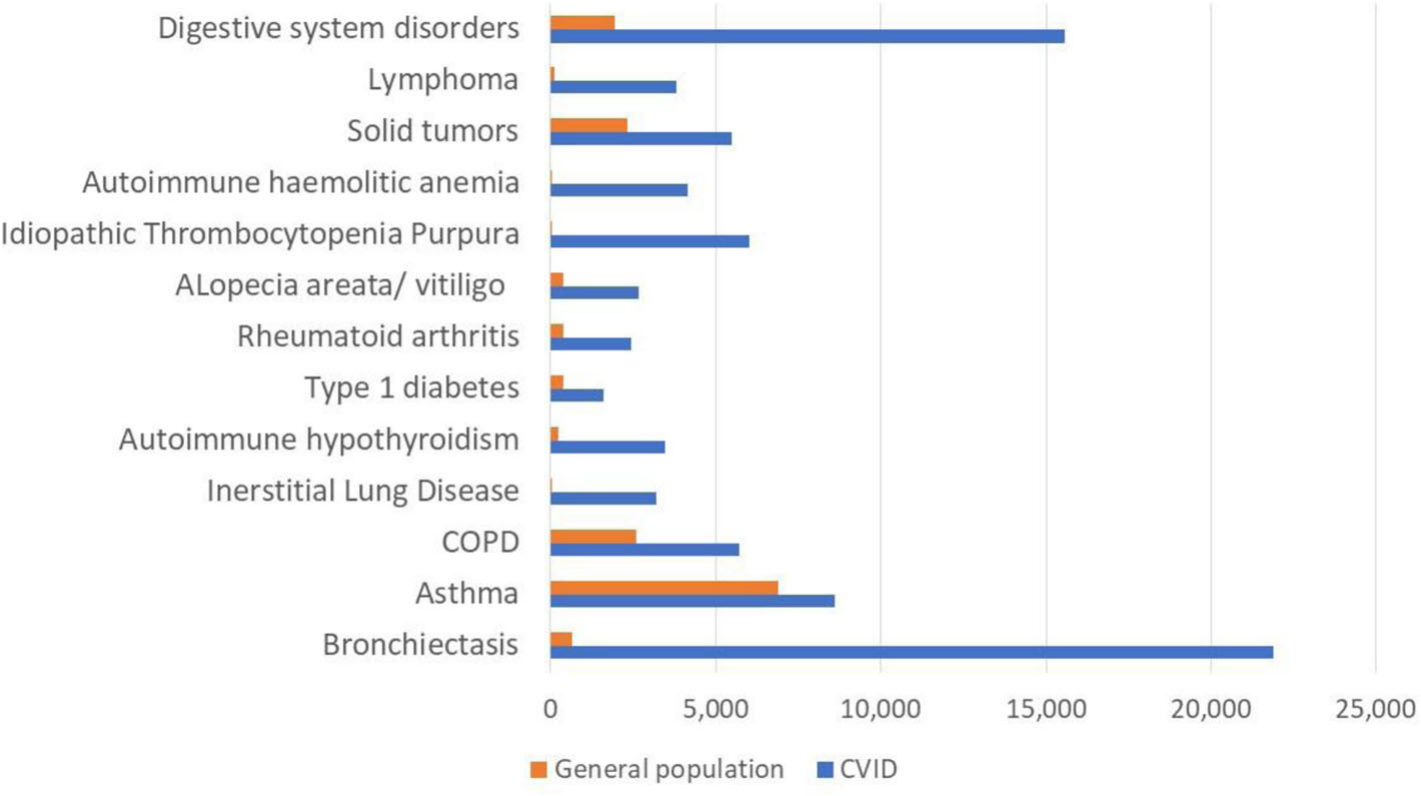
Prevalence of non-communicable comorbidities. Average annual age-standardized prevalence rate per 100,000 over the period 2004–2014. CVID cohort versus general population*. All ages, both sexes. *Source: Global Burden of Disease Studies, Western Europe: http://ghdx.healthdata.org/gbd-results-tool

Chronic lung disease was most common, with an average annual age-standardized prevalence of bronchiectasis of 21.9% (20.1; 23.8), asthma: 8.6% (7.7; 9.6), COPD: 5.7% (5.1; 6.3) and GLILD: 3.2% (2.5; 3.8). These prevalence rates were higher than in the general population by a factor of 65.3 in GLILD, 34.0 in bronchiectasis, and 2.2 and 1.3 for COPD and asthma, respectively (Fig. 4 and Additional file 4).

The age-standardized prevalence of autoimmune disorders was 23.2%. Autoimmune cytopenias were dominated by idiopathic thrombocytopenia purpura (ITP) in 6.0% (5.3; 6.8) and autoimmune hemolytic anemia in 4.1% (3.7; 4.7). Overall, the prevalence of autoimmune cytopenias was 702.9 times higher than in the general population. Among the organ and systemic autoimmunities, hypothyroidism was the most prevalent type: 3.5% (3.1; 3.9), followed by alopecia areata and vitiligo: 2.7% (2.4; 2.9), rheumatoid arthritis: 2.4% (2.2; 2.7) and type 1 diabetes: 1.6% (1.4; 1.7). Twenty-six percent of patients had another type of autoimmunity, mostly unspecified. Compared to the general population, the overall prevalence of autoimmunity was 7.6 times higher in the CVID patients.

Digestive system disorders were annually occurring in 15.6% (13.9; 17.6) of patients, exceeding the prevalence rate in the general population by a factor of 8.1. Of these, 60.9% had enteropathy comprising non-infective gastroenteritis and/or colitis, coeliac disease, Crohn’s disease, malabsorption and functional diarrhea.

Annual age-standardized prevalence of solid tumors was 5.5% (4.7; 6.2) with skin cancer being the most common type and accounting for 30.8% of all solid tumors, followed by breast cancer (12.2%) and lung cancer (7.5%). Gastric cancer was registered in 1.0% of the cohort over the observation period, an 8.6 times higher prevalence compared to the European population [44]. Lymphoma annually occurred in 3.8% (3.2; 4.4). The prevalence of lymphoma and all solid cancers exceeded the prevalence rates in the general population by a factor of 32.5 and 2.4, respectively.

The mean annual age-standardized prevalence of splenomegaly was 19.0%, granuloma (other than GLILD) 4.4%, and lymphoproliferation 3.9%. Blood disorders (other than autoimmune cytopenias) were registered at least once in 14.5% during the follow-up period, with about 50% of cases attributable to anemia and thrombocytopenia.

**Fig. 5:**
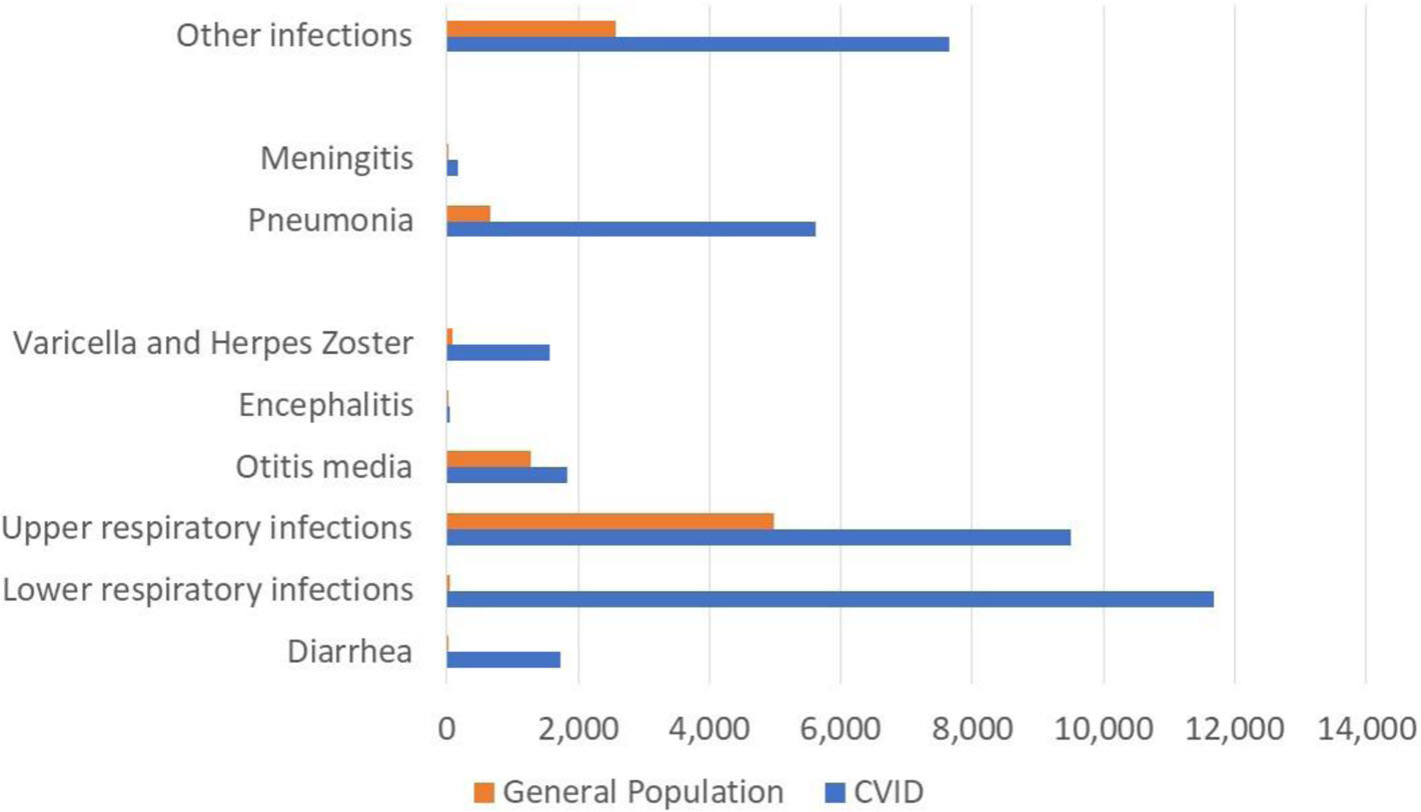
Prevalence of infections. Average annual age-standardized prevalence rate per 100,000 over the period 2004–2014. CVID cohort versus general population*. All ages, both sexes. *Source: Global Burden of Disease Studies, Western Europe: http://ghdx.healthdata.org/gbd-results-tool

Serious bacterial infections (SBIs) had a much higher annual prevalence in the CVID cohort than in the general population. Pneumonia occurred in 5.6% (4.9; 6.4), meningitis in 0.17% (0.05; 0.4) of CVID patients, exceeding the respective prevalence in the general population by factors 8.5 and 76.2 (Fig. 5). The rates of pneumonia and meningitis per person-year were 0.06 (0.05–0.07) and 0.002 (0.0009–0.004) respectively. The annual prevalence of other types of infections – lower and upper respiratory, otitis, varicella, herpes zoster, diarrhea etc. – was 34.0% (29.8; 38.7). The overall infection rate per person-year including SBIs was 0.4 (0.38; 0.41) (Additional file 5).

The annual age-standardized YLD rate associated with the comorbidities of CVID summed up to 8772 (6069; 12,363) per 10^5^ of this CVID cohort. Infections had the largest contribution to the disability related health loss: 32.7%, followed by autoimmunity: 23.1%, chronic lung diseases: 22.2%, digestive system disorders: 13.7% and neoplasms: 8.2%. Nearly half (44%) of the disability burden was attributable to infections and bronchiectasis (Additional file 4).

### Disability adjusted life years

The individual burden of CVID, i.e. the annual mean age-standardized DALY rate per 10^5^ of this cohort, was 36,785 (33,078; 41,380) (Table 3).

**Table 3:** Mean annual age-standardized YLLs, YLDs and DALYs over the period 2004–2014

	Mean (95% CI) annual age-standardized rates per 100,000 of population	
Attribute of health loss	CVID cohort, ESID registry	General population, Western Europe
Years of Life Lost to death	28,013 (27,009; 29,017)	9314 (9296; 9332)
Years of Life Lost to disability	8772 (6069; 12,363)	1196 (751; 1715)
Disability-Adjusted Life Years	36,785 (33,078; 41,380)	10,510 (9998; 10,759)

Accounting for the prevalence of CVID in Europe, estimated at 1 in 25,000 people [5, 6], the annual population health loss associated with CVID, i.e. societal disease burden, comprised 1.5 (1.3; 1.7) DALYs per 10^5^ of the European population.

**Fig. 6:**
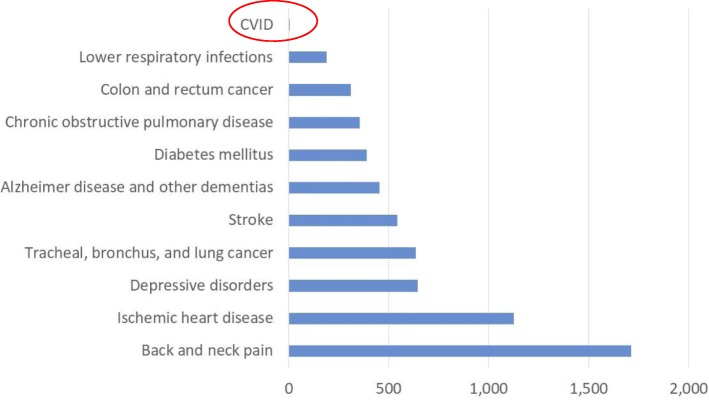
Burden of disease to society: CVID versus top-ten health problems in Europe. Annual age-standardized DALY rate per 100,000 of general population*. *Source: Global Burden of Disease Studies, Western Europe 2015: http://ghdx.healthdata.org/gbd-results-tool

**Fig. 7:**
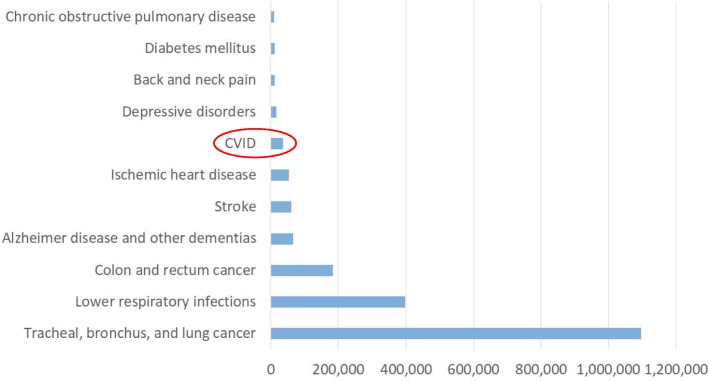
Burden of disease to individual patient: CVID versus top-ten health problems in Europe. Annual age-standardized DALY rate per 100,000 of diagnosed population*. *Calculated as DALY per 100,000 general population X 100,000/ disease prevalence per 100,000 general population

The ten leading health problems in Europe identified by the GBD caused a mean societal burden of between 187 (lower respiratory infections) and 1712 (back and neck pain) DALY per 10^5^ of general population [24] (Fig. 6 and Additional file 6). The burden of these diseases to the individual patient, i.e. estimated as a mean DALY rate per 10^5^ of population diagnosed with a respective disease, varied between 10,445 (chronic obstructive pulmonary disease); and 1,096,432 (tracheal, bronchus and lung cancers). The individual burden of CVID was somewhat below the individual burden of stroke and ischaemic heart disease: 60,247 and 52,953 DALY respectively; and considerably higher than the burden of depressive disorders, diabetes mellitus and COPD: 16,710; 12,043; and 10,445 DALY respectively (Fig. 7 and Additional file 6).

### Explorative analysis of the risk factors of health loss

The overall survival rate from the year of the diagnosis was 0.95 (0.93; 0.97) at 10 years, 0.76 (0.71; 0.81) at 25 years, and 0.49 (0.37; 0.66) at 45 years follow-up (Additional file 7). Increased mortality was associated with solid tumor, HR (95% CI): 2.69 (1.10; 6.57) *p* = 0.030, lymphoma: 5.48 (2.36; 12.71) *p* < .0001 and GLILD: 4.85 (1.63; 14.39) *p* = 0.005 (Table 4). Other factors associated with increased mortality were parental consanguinity: 4.42 (1.66; 11.75) *p* = .003, higher age at symptoms onset: 1.04 (1.03; 1.05) p < .0001, higher age at CVID diagnosis: 1.04 (1.03; 1.05) p < .0001, and diagnostic delay adjusted for the age at symptoms onset: 1.04 (1.02; 1.06) *p* = .0003. No association between survival and sex, Ig replacement dose, or diagnostic delay adjusted for the age of the CVID diagnosis was found (Table 5).

**Table 4:** Association between comorbidities and all-cause mortality. Results of Cox proportional hazard model with comorbidities as time-dependent covariate (*N* = 972)

Comorbidity	HR (95% CI)^a^	*P*-Value	HR (95% CI)^b^	*P*-Value
Bronchiectasis	0.84 (0.44; 1.62)	0.612	0.83 (0.40; 1.86)	0.633
Splenomegaly	2.11 (1.17; 3.82)	0.013	1.67 (0.82; 3.39)	0.155
Autoimmunity (organ/ systemic)	1.61 (0.79; 3.27)	0.187	1.52 (0.67; 3.43)	0.311
Autoimmune cytopenia	0.72 (0.22; 2.35)	0.591	1.08 (0.33; 3.57)	0.897
Enteropathy	1.39 (0.54; 3.56)	0.493	0.97 (0.28; 3.41)	0.962
Solid tumor	3.19 (1.55; 6.57)	0.002	2.69 (1.10; 6.57)	0.030
Lymphoma	3.95 (1.81; 8.66)	0.001	5.48 (2.36; 12.71)	<.0001
GLILD	3.80 (1.47; 9.85)	0.006	4.85 (1.63; 14.39)	0.005

^a^univariable analysis

^b^adjusted for age of CVID symptoms onset

**Table 5:** Results of an explorative survival risk factor analysis. Results are obtained with Cox proportional hazards model (*N* = 2700)

Risk factor	HR: Mean (95% CI)	*P* value
Age at diagnosis^a^	1.04 (1.03; 1.05)	<.0001
Age at symptoms onset^a^	1.04 (1.03, 1.05)	<.0001
Sex (female)^a^	1.14 (0.77; 1.69)	0.515
Age at diagnosis^b^	1.05 (1.04–1.06)	<.0001
Age at symptoms onset^b^	1.05 (1.04–1.06)	<.0001
Consanguinity^c^	4.42 (1.66; 11.75)	0.003
Monthly Ig dose^c^	1.00 (0.99; 1.00)	0.780
Diagnostic delay^d^	0.99 (0.97–1.01)	0.221
Diagnostic delay^e^	1.04 (1.02; 1.06)	0.0003

^a^resuls of a univariable analysis

^b^results adjusted for diagnostic delay

^c^results stratified for the age at CVID diagnosis and sex

^d^results adjusted for the age at CVID diagnosis

^e^results adjusted for the age of symptoms onset

### Diagnostic delay

Diagnostic delay adjusted for the age at CVID symptoms onset was associated with the prevalence of bronchiectasis: HR (95% CI): 1.03 (1.01; 1.04) *p* = .0001, solid tumor: 1.08 (1.04; 1.11) p < .0001, and enteropathy: 1.02 (1.00; 1.05) *p* = .0447. Diagnostic delay adjusted for the age of CVID diagnosis was associated with the prevalence of bronchiectasis only: 1.01 (1.00; 1.03) *p* = 0.0472 (Table 6). A comparison of three consecutive time periods of CVID diagnosis (≤ 1980; 1981–1999; and ≥ 2000) revealed no significant difference in diagnostic delay (independent sample Kruskal-Wallis test, *p* = .228) (Table 7).

**Table 6:** Association between diagnostic delay and the prevalence of comorbidities in the CVID cohort. Results of proportional Cox regression (*N* = 972)

Comorbidities	HR: Mean (95% CI)			
	Results adjusted for the age at CVID diagnosis	*P* value	Results adjusted for the age at symptoms onset	*P* value
Bronchiectasis	1.01 (1.00; 1.03)	*0.0472*	1.03 (1.01; 1.04)	*0.0001*
Solid tumor	1.01 (0.99; 1.04)	*0.2604*	1.08 (1.04; 1.11)	*< 0.0001*
Lymphoma	0.96 (0.91; 1.02)	*0.1919*	0.98 (0.93; 1.04)	*0.5713*
Splenomegaly	1.00 (0.98; 1.02)	0.9053	1.00 (0.98; 1.02)	0.9611
Chronic lung disease (COPD, asthma)	0.98 (0.96; 1.01)	0.2543	0.99 (0.96; 1.02)	0.4545
Autoimmunity (organ, systemic)	1.00 (0.98; 1.03)	0.9725	1.00 (0.98; 1.03)	0.6958
Enteropathy	1.02 (1.00; 1.04)	0.1206	1.02 (1.00; 1.05)	0.0447
Autoimmune cytopenia	0.97 (0.92; 1.02)	0.1973	0.96 (0.91; 1.01)	0.0892
GLILD	0.90 (0.80; 1.01)	0.0771	0.91 (0.80; 1.02)	0.1045
Granuloma (other than GLILD)	0.98 (0.92; 1.03)	0.3726	0.97 (0.92; 1.03)	0.3362
Lymphoproliferation	0.98 (0.93; 1.03)	0.4132	0.99 (0.94; 1.04)	0.6758

**Table 7:** Diagnostic delay per period of diagnosis (years)

Period of diagnosis	Mean 95% CI	Median	Range
≤ 1980	7.4 (5.1; 9.8)	3.5	0–44
1981–1999	8.6 (7.7; 9.5)	4.0	0–65
≥ 2000	8.8 (8.2; 9.3)	4.0	0–69

## Discussion

### Burden of disease

The burden of more than 300 conditions worldwide has been quantified by the GBD project, however, the burden of many rare diseases remains unknown. This study presents the first estimation of the burden of CVID in Europe based on the data of the ESID registry, the largest Primary Immunodeficiency registry in the world. The annual loss of healthy life years due to premature death and living with disability was estimated between 33,078 and 41,380 per 100,000 in the CVID population and corresponded with 1.3 to 1.7 disability-adjusted life years per 100,000 in the general population in Europe.

Due to the low prevalence of CVID, the societal burden of this rare immune disorder is not comparable to that of common conditions identified by the GBD as the leading causes of health loss in Europe, such as ischemic heart disease or diabetes that annually cause a respective loss of 1125 and 389 DALY per 100,000 population in Western Europe [24]. However, the burden to the individual CVID patient is comparable with the individual burden of stroke or ischemic heart disease, and even substantially higher than the individual disease burden to patients with diabetes mellitus or COPD. In the CVID cohort, loss of healthy life years due to premature death was three times higher than in the general population. Loss of healthy life years due to comorbidities and infections was 7.3 times higher in the CVID cohort than years of life lost annually due to the same diseases in the general population.

These findings challenge the current approach to the prioritization of the healthcare problems based on the burden of a disease to the society, as rare diseases are likely to be discriminated due to their low prevalence and relatively modest impact on population health. Estimating the burden of disease to the individual patient should serve as an important additional guidance for the decisions on public health priorities and resource allocation in research and clinical care. Currently, more than 7000 rare diseases have been known affecting 30 to 40 million people in Europe, with only about 1% having an adequate treatment, while the burden of these diseases is largely unknown [28].

Poorer survival in CVID was associated with the prevalence of solid tumor, lymphoma and GLILD, showing consistency with the results of some large cohort studies [30, 45]. Our analysis of disability burden adds to this knowledge that despite the Ig replacement therapy nearly half of the total disability in the CVID cohort was attributable to infections and bronchiectasis, a frequent chronic complication of recurrent lower respiratory infections [29]. This finding emphasizes the importance of an adequate Ig replacement dosing. While no universal guidelines for an optimal Ig dose exist, current evidence suggests individualization of the Ig dose to attain infection-free outcomes [17]. In view of a relatively high prevalence of SBIs, e.g. pneumonia had a 8.5 times higher prevalence rate compared to the general population, the question occurs whether the administered Ig replacement regimens - the mean dose was overall below 500 mg/kg - were optimal for each individual patient. This study was not designed to establish a causative relationship between the drug dose and the clinical outcomes; moreover, some relevant information on potential confounders was missing, e.g. patient compliance to the therapy, or the prescribed antibiotics regimen. However, a recent meta-analysis by Orange et al. showed that the incidence of pneumonia with maintenance of 500 mg/dL IgG trough level (0.113 cases per patient-year) was 5-fold that with 1000 mg/dL (0.023 cases per patient-year), declining by 27% with each 100 mg/dL increment in trough IgG level; and demonstrated a linear relationship between the trough IgG levels and the Ig dose: an increase by 121 mg/dL with each incremental increase of monthly Ig dose by 100 mg/kg [16].

The mortality rate was four times higher in patients with parental consanguinity, suggesting unidentified autosomal recessive disease underlying the CVID-classification in these patients. Parental consanguinity was previously reported as a predictor of death in PID [37–40]. Higher age at symptoms onset and higher age at CVID diagnosis were associated with poorer survival chances, a confirmation of previous findings [11, 30, 31]. We also explored diagnostic delay in a bivariable survival analysis, first in conjunction with the age at diagnosis, then with the age at symptoms onset. The first analysis shows whether/ how diagnostic delay affects survival in CVID patients diagnosed at the same age, the second – how it affects those patients who experienced the symptoms onset at the same age. Our analysis has shown that diagnostic delay - when accounting for the age at symptoms onset - is a predictor of mortality and comorbidities. Each year of increase in diagnostic delay was associated with an increase of the risk of death by 4%, bronchiectasis by 3%, solid tumor by 8% and enteropathy by 2%. Adjusting for the age of symptoms onset rather than for the age of diagnosis may be clinically more relevant as diagnostic delay of several years is frequent and rather reflects the healthcare system (in)efficiency rather than the clinical marker of the disease. These findings are hypothesis generating and need to be confirmed in prospective studies. A diagnostic delay of ≥1 (up to 69!) years was found in 84% of the cohort and therefore represents a major concern, especially in view of our finding that the length of this delay has not decreased over a period of decades, despite the efforts of the primary immunodeficiency (PID) community to facilitate timely diagnosis of immunodeficiencies e.g. through a system of warning signs and educational activities. An effective algorithm based on the use of electronic patient health records to support non-expert primary and secondary care physicians to identify potential PID is not yet available and has to be developed with high priority [32–35]. It’s been estimated that the treatment cost of undiagnosed PID patients in the U.S was 5 times higher than of those diagnosed and receiving Ig replacement therapy [36]. Introducing tools supporting early recognition of potential CVID could deliver a high return on investment.

### Limitations of the study

Comorbidities and infections were registered in 36.0 and 26.3% of patients respectively; and our prevalence estimation was based on these subsets. To address the uncertainty associated with the quality of the registry data, the results were compared with previously published findings. The survival rate as well as the period prevalence of comorbidities and SBIs were consistent with those reported elsewhere [2, 3, 6, 14–16, 29–31, 41–43]. However, the prevalence of infections, particularly other than SBIs, was probably underreported, as the overall infection rate of 0.4 per person-year was lower than that reported by Lucas et al. and by Berger in patients treated with immunoglobulin: a rate of 2.16 and 2.8 to 5.2 per person-year respectively [15, 42]. Walsh and colleagues observed a decline from a median of 2.0 infections per pretreatment year to 0.4 infections per year posttreatment, referring to sinopulmonary infections only [43]. This assumed underreporting of infections may have caused underestimation of the true disability associated with infections in CVID.

For the estimation of years lost to disability, we used the results of the GBD study for YLDs in the general population, adopting the assumption that the severity distribution in CVID patients would be comparable with that observed in the general population. A lack of definition and quantification of the burden of some CVID comorbidities in the scientific literature, such as unspecified autoimmunity and certain blood disorders, may have led to additional underestimation of the total CVID disability. A structural and uniform collection of the self-perceived health status in patients with CVID would help to better determine the burden for CVID patients with different clinical phenotypes and has been introduced by individual centers [46, 47].

A division of the cohort in clinically relevant subsets for the purpose of a comparative burden of disease analysis was not feasible in this study, since results of genetic tests and the available immunological measurements, such as T-lymphocytes counts, were too limited in terms of their quantity and/or quality. The observed high mortality in children, and the reported cases of parental consanguinity are in general not typical for CVID and may indicate that patients with Combined Immunodeficiency might have been classified as CVID in some cases [48–50].

The records of the ESID registry used in this study are not necessarily representative for the national clinical practices in Europe, as a positive self-selection of the contributing treatment centers is possible, resting on the individual commitment of a select group of clinical immunologists. Furthermore, there was a strong variation in the number of patients and the registration rate per country, inhibiting a meaningful between-country comparison. A further specification of the burden of CVID per country is warranted based on the national primary immunodeficiency registries.

As previously discussed, at the moment, different centres classify patients in different ways: some only accept a diagnosis of CVID in case both IgG and IgA are low, others consider decreased IgG and IgM also sufficient. Also, many hypogammaglobulinemic patients who do not fully meet the diagnostic criteria for CVID show a severe course with infections and bronchiectasis. A regular repetition of burden of disease studies based on a more extended datasets containing genetic and immunolaboratory tests is recommended, to further identify pheno- and genotypes responsible for a higher morbidity and mortality, to track the evolution of care standards and clinical outcomes over time and to compare the results of the healthcare systems in different regions of the world.

## Conclusion

The rates of mortality and serious comorbidities of people with CVID drastically exceed the respective rates in the general population, imposing a high disease burden to the individual patient. Our study demonstrates the need to advance timely diagnosis and treatment of CVID, to achieve improved clinical outcomes and reduce the burden of disease. The importance of a consistent and uniform data registration on PID patients in Europe, to improve understanding of these rare heterogeneous diseases, cannot be overemphasized.

## Additional files

Additional file 1: Overview of the ESID registry data used in this study. (DOCX 14 kb)

Additional file 2: Table: Rates of death and Years of Life Lost to all-cause mortality in the CVID cohort and the general population, per 5-year age interval. (DOCX 17 kb)

Additional file 3: Table: Prevalence rates of CVID comorbidities. (DOCX 15 kb)

Additional file 4: Table: Age standardized rate of prevalence of comorbidities and Years Lost to Disability in the CVID cohort and the general population. (DOCX 27 kb)

Additional file 5: Table: Infection rates in the CVID cohort. (DOCX 22 kb)

Additional file 6: Burden of disease to society and the individual patient. (DOCX 15 kb)

Additional file 7: All-cause mortality table, CVID cohort. (DOCX 16 kb)

Additional file 8: Diagnostic criteria of CVID. (DOCX 15 kb)

## Abbreviations

CI: Confidence interval
COPD: Chronic obstructive pulmonary disease
CVID: Common variable immunodeficiency disorders
DALY: Disability adjusted life years
ESID: European Society for Immunodeficiencies
GBD: Global burden of disease study
GLILD: granulomatous-lymphocytic interstitial lung disease
HR: Hazard ratio
ICD-10: International classification of diseases
ITP: Idiopathic thrombocytopenia purpura
PPTA: Plasma Protein Therapeutics Association
SBIs: Serious bacterial infections
WHO: World Health Organization
YLD: Years lost to disability
YLL: Years of life lost to premature death

## Footnotes

1 Today, there is no complete consensus regarding the definition of CVID. The discussion particularly concerns the presence of a decreased IgA level as an imperative diagnostic criterion. While ESID recommends diagnosis of CVID based on a marked decrease of IgG *and* a marked decrease of IgA (with or without low IgM levels; 2014), the international consensus “ICON” (2016) suggests less stringent diagnostic criteria: a marked decrease of IgG and at least one of IgM *or* IgA (3,7, Additional file 8). Patient inclusion was based solely on the diagnosis by their immunologists and not verified according to the diagnostic criteria due to limitations of the dataset.
