# Supplementary material for: The burden of common variable immunodeficiency disorders: a retrospective analysis of the European Society for Immunodeficiency (ESID) registry data
Source: Orphanet J Rare Dis. 2018 Nov 12;13:201. doi: 10.1186/s13023-018-0941-0 (PMC6233554; doi:10.1186/s13023-018-0941-0)
Supplement: Supplementary file 1 — Overview of the ESID registry data used in this study. (DOCX 14 kb) [file 13023_2018_941_MOESM1_ESM.docx]

Additional file 1: Overview of the ESID registry data used in this study

Patient information

1. ID number
2. Country
3. Sex
4. Date of birth
5. Status (alive/dead)
6. Familial case
7. Consanguinity
8. Date of death
9. Date of clinical CVID diagnoses
10. Date of onset of symptoms
11. Weight/ Weight measurement date

Immunoglobulin replacement therapy

1. Route of administration: IMIG/IVIG/SCIG
2. Place of administration: Home/ Hospital
3. Start date
4. Stop date
5. Reason stopped
6. Side effects
7. Dose, value
8. Dose, unit
9. Relative dose (per body weight), value
10. Relative dose (per body weight), unit
11. Dose, frequency
12. Dose, interval
13. Compliance

Concomitant diseases

1. ICD 10 code
2. ICD 10 text
3. Date of diagnosis

Infection history

1. ICD 10 code
2. ICD 10 text
3. Start date
4. Stop date

Other data*:

- Antibiotics and other medications: Drug; Dose; Dose interval; Route of administration; Start date; Stop date; Side effects; Reason stopped; Compliance
- Laboratory tests: Date; Value; Unit (Leukocytes; Thrombocytes; Erythrocytes; Hemoglobin; Lymphocytes; Neutrophils; Eosinophils; Basophils; Monocytes; IgG Subclasses; TB cell panel; CD19+/CD27+; CD20+/CD27+; NK-T cells; CRP)
- Immunizations: Date; Vaccine
- Adverse events: Date of onset; Date of resolution; Severity; Action; Result; Suspected adverse drug; Organ; Type of reaction
- Self-reported outcomes: Days in hospital; Days missed from school/work; Number of infectious episodes; Number of serious bacterial infections; Visit date; Last visit date

*Due to low registration rate and/or insufficient quality, these data could only be used to elicit the dates of the patient visits to the immunology centre
