# Supplementary material for: The burden of common variable immunodeficiency disorders: a retrospective analysis of the European Society for Immunodeficiency (ESID) registry data
Source: Orphanet J Rare Dis. 2018 Nov 12;13:201. doi: 10.1186/s13023-018-0941-0 (PMC6233554; doi:10.1186/s13023-018-0941-0)
Supplement: Supplementary file 2 — Table: Rates of death and Years of Life Lost to all-cause mortality in the CVID cohort and the general population, per 5-year age interval. (DOCX 17 kb) [file 13023_2018_941_MOESM2_ESM.docx]

### Additional file 2. Table: Rates of death and Years of Life Lost to all-cause mortality in the CVID cohort and the general population, per 5-year age interval

| Death rate/100.000 (95% CI) | | | **Years of Life Lost/100.000 (95% CI)** | |
| --- | --- | --- | --- | --- |
| Age group (years) | **CVID cohort*** | **General population** | **CVID cohort*** | **General population** |
| 5-9 | 298 (82; 1,080) | 9.3 (9.2; 9.4) | 22,131 (21,930; 22,332) | 738 (729; 747) |
| 10-14 | 440 (171; 1,125) | 10.7 (10.6; 10.8) | 31,165 (30,721; 31,609) | 792 (787; 798) |
| 15-19 | 276 (94; 809) | 30.6 (30.5; 30.8) | 18,407 (18,182; 18,632) | 2,118 (2,110; 2,128) |
| 20-24 | 384 (131; 1,122) | 44.9 (44.7; 45.1) | 23,286 (23,082; 23,491) | 2,888 (2,878; 2,900) |
| 25-29 | 450 (175; 1,151) | 48.9 (48.7; 49.0) | 26,220 (25,993; 26,448) | 2,904 (2,896; 2,914) |
| 30-34 | 540 (231; 1,258) | 59.9 (59.8; 60.1) | 27,268 (26,957; 27,589) | 3,261 (3,253; 3,270) |
| 35-39 | 301 (102; 880) | 85.7 (85.5; 85.9) | 13,537 (13,270; 13,804) | 4,239 (4,229; 4,249) |
| 40-44 | 694 (352; 1,364) | 136.3 (136.0; 136.6) | 28,316 (27,812; 28,820) | 6,073 (6,061; 6,087) |
| 45-49 | 1,192 (698; 2,028) | 225.7 (225.2; 226.2) | 42,236 (41,526; 42,947) | 8,976 (8,958; 8,995) |
| 50-54 | 1,075 (567; 2,031) | 362.7 (362.0; 363.4) | 35,699 (35,360; 36,038) | 12,719 (12,694; 12,745) |
| 55-59 | 556 (216; 1,420) | 551.4 (550.3; 552.5) | 15,014 (14,908; 15,120) | 16,784 (16,752; 16,818) |
| 60-64 | 1,583 (886; 2,812) | 829.9 (828.3; 831.4) | 35,727 (35,413; 36,040) | 21,488 (21,449; 21,528) |
| 65-69 | 1,639 (865; 3,086) | 1,265 (1?263; 1,267) | 30,346 (30,187; 30,505) | 27,149 (27,100; 27,198) |
| 70-74 | 2,813 (1,578; 4,967) | 2,043 (2,040; 2,047) | 47,008 (46,561; 47,451) | 35,126 (35,062; 35,190) |
| 75-79 | 3,756 (1,915; 7,235) | 3,513 (3,507; 3,519) | 46,995 (46,776; 47,215) | 46,495 (46,413; 46,579) |
| 80+ | 5,747 (2,480; 12,757) | 10,289 (10,274; 10,303) | 61,639 (61,793; 61,886) | 63,054 (62,968; 63,139) |
| Standardized** | 865 (678; 1,052) | 519 (518; 520) | 28,013 (27,009; 29,017) | 9,314 (9,296; 9,332) |

*Calculated based on the data of 2,700 patients from the ESID registry cohort included in the Burden of CVID analysis

**Standardized using the WHO world population standard (21)
