# Supplementary material for: The burden of common variable immunodeficiency disorders: a retrospective analysis of the European Society for Immunodeficiency (ESID) registry data
Source: Orphanet J Rare Dis. 2018 Nov 12;13:201. doi: 10.1186/s13023-018-0941-0 (PMC6233554; doi:10.1186/s13023-018-0941-0)
Supplement: Supplementary file 3 — Table: Prevalence rates of CVID comorbidities. (DOCX 15 kb) [file 13023_2018_941_MOESM3_ESM.docx]

### Additional file 3. Table: Prevalence rates of CVID comorbidities

| CVID comorbidity | Previously reported  period prevalence* | ESID registry:  period prevalence** | ESID registry:  annual crude prevalence | ESID registry:  annual age-standardized prevalence*** |
| --- | --- | --- | --- | --- |
| Bronchiectasis | 25% (2)  29% (44)  11.2% (30) | 26.8% | 23.7% | 21.9% |
| Splenomegaly | 30% (2)  26% (3) 22.4% (6) 26.4% (44) | 24.0% | 20.9% | 19.0% |
| Autoimmunity | 28.6% (30) 25-30% (3) 25.9% (44) | 25.5% | 22.8% | 23.2% |
| Solid tumor | 7% (30) | 8.2% | 6.9% | 5.5% |
| Lymphoma | 3.0% (2)  8.2% (30) 6.7% (3) | 5.9% | 4.5% | 3.8% |
| Any neoplasm | 6.0% (2)  15.2% (30) 6-9% (3) | 14.1% | 11.4% | 9.3% |
| Enteropathy | 9% (2)  9% (3) | 9.9% | 9.6% | 9.5% |
| Granuloma | 8.0% (2)  9.7% (30) 8-22% (3) 3.5% (6) | 5.2% other than GLILD  3.9% GLILD  All granulomas: 9.1% | 4.9% | 4.4% other than GLILD  3.2% GLILD  All granulomas: 7.6% |

*Reported crude prevalence rates observed in large CVID cohorts over observation periods varying between a mean of 3.8 and 25.6 years

**Crude period prevalence calculated from the ESID registry subset of 972 (36.0%) patients with registered comorbidities over a median follow-up period of 6 years

***Standardized using the WHO world population standard (21)
