# Supplementary material for: The burden of common variable immunodeficiency disorders: a retrospective analysis of the European Society for Immunodeficiency (ESID) registry data
Source: Orphanet J Rare Dis. 2018 Nov 12;13:201. doi: 10.1186/s13023-018-0941-0 (PMC6233554; doi:10.1186/s13023-018-0941-0)
Supplement: Supplementary file 4 — Table: Age standardized rate of prevalence of comorbidities and Years Lost to Disability in the CVID cohort and the general population. (DOCX 27 kb) [file 13023_2018_941_MOESM4_ESM.docx]

Additional file 4. Table: Age standardized rate of prevalence of comorbidities and Years Lost to Disability in the CVID cohort and the general population

| Concomitant diseases of CVID | Age-standardized prevalence, Rate per 100,000 (95%CI) | | Years Lost to Disability, Rate per 100,000 (95%CI) | | |
| --- | --- | --- | --- | --- | --- |
| **Non-communicable diseases*** | CVID | General population | CVID* | General population |  |
| *Chronic lung disease* |  |  |  |  |  |
| Bronchiectasis | 21,904 (20,055; 23,753) | 645 (568; 729) | 978 (637; 1,382) | 29 (18; 42) |  |
| Asthma | 8,614 (7,670; 9,558) | 6,873 (6,244; 7,529) | 385 (243; 555) | 307 (198; 437) |  |
| COPD | 5,694 (5,070; 6,318) | 2,574 (2,368; 2,772) | 203 (162; 249) | 92 (76; 109) |  |
| Interstitial Lung Disease | 3,200 (2,500; 3,800) | 49 (45; 53) | 380 (204; 591) | 6 (4; 8) |  |
| *Autoimmunity (organ / systemic)* |  |  |  |  |  |
| Autoimmune hypothyroidism | 3,473 (3,056; 3,924) | 215 (189; 243) | 66 (58; 75) | 4 (4; 5) |  |
| Type 1 diabetes | 1,586 (1,417; 1,742) | 363 (336; 391) | 123 (82; 171) | 28 (19; 38) |  |
| Rheumatoid arthritis | 2,440 (2,180; 2,680) | 389 (358; 426) | 575 (388; 773) | 92 (64; 123) |  |
| Alopecia areata / vitiligo | 2,684 (2,398; 2,948) | 379 (359; 399) | 91 (53; 141) | 13 (8; 19) |  |
| *Autoimmune cytopenia* |  |  |  |  |  |
| Idiopathic Thrombocytopenia Purpura | 6,032 (5,308; 6,816) | 10 (8; 11) | 959 (844; 1,084) | 1.5 (1.3; 1.7) |  |
| Autoimmune haemolytic anaemia | 4,160 (3,660; 4,700) | 5 (4; 6) | 216 (190; 244) | 0.3 (0.2; 0.3) |  |
| *Neoplasms* |  |  |  |  |  |
| Solid tumors | 5,500 (4,700; 6,200) | 2,337 (2,091; 2,741) | 448 (300; 592) | 191 (133; 262) |  |
| Lymphoma | 3,800 (3,200; 4,400) | 117 (102; 130) | 274 (186; 382) | 8 (6; 11) |  |
| *Digestive system disorders* | 15,571 (13,681; 17,550) | 1,930 (1,892; 1,968) | 1,205 (771; 1,765) | 149 (107; 198) |  |
| ***Serious Bacterial Infections***** |  |  |  |  |  |
| Pneumonia | 5,621 (4.875; 6,449) | 661 (117; 1,1160) | 1,293 (1,123; 1,483) | 152 (39; 267) |  |
| Meningitis | 174 (53; 403) | 2 (2; 3) | 322 (76; 897) | 4.2 (3.0; 5.5) |  |
| ***Other infections***** |  |  |  |  |  |
| Lower respiratory infections | 11,671 (10,619; 12,792) | 42 (40; 44) | 702 (451; 1,035) | 2.5 (1.7; 3.5) |  |
| Upper respiratory infections | 9,504 (8,544; 10,539) | 4,975 (4,443; 5,523) | 112 (62; 188) | 59 (32; 99) |  |
| Otitis media | 1,827 (1,416; 2,326) | 1,277 (1,144; 1,428) | 50 (27; 86) | 35 (22; 53) |  |
| Diarrhoea | 1,740 (1,380; 2,180) | 25 (24; 26) | 284 (159; 480) | 4.0 (2.8; 5.6) |  |
| Encephalitis | 39 (11; 141) | 8 (6; 11) | 5 (1; 16) | 1.0 (0.7; 1.3) |  |
| Varicella and Herpes Zoster | 1,570 (1,189; 2,041) | 86 (81; 92) | 55 (27; 102) | 3.0 (1.9; 4.6) |  |
| Other infections | 7,649 (6,688; 8,696) | 2,581 (820; 3,407) | 46 (33; 53) | 15 (11; 22) |  |
| **TOTAL Years lost to disability** | | | 8,772 (6,069; 12,363) | 1,196 (751; 1,715) |  |

*Calculated from the ESID registry subset of 972 (36.0%) patients with registered comorbidities

**Calculated from the subset of 710 (26.3%) patients with registered infections
