# Supplementary material for: The burden of common variable immunodeficiency disorders: a retrospective analysis of the European Society for Immunodeficiency (ESID) registry data
Source: Orphanet J Rare Dis. 2018 Nov 12;13:201. doi: 10.1186/s13023-018-0941-0 (PMC6233554; doi:10.1186/s13023-018-0941-0)
Supplement: Supplementary file 5 — Table: Infection rates in the CVID cohort. (DOCX 22 kb) [file 13023_2018_941_MOESM5_ESM.docx]

Additional file 5. Table: Infection rates in the CVID cohort

| **Type infection** | **Infections per year (95% CI)*** |
| --- | --- |
| *Serious bacterial infections* |  |
| Pneumonia | 0.0574 (0.0501; 0.0656) |
| Meningitis | 0.0021 (0.0009; 0.0043) |
| *Other infections* |  |
| Lower respiratory | 0.1362 (0.1255; 0.1475) |
| Upper respiratory | 0.1044 (0.0951; 0.1143) |
| Otitis media | 0.0157 (0.0121; 0.02) |
| Varicella and herpes zoster | 0.0161 (0.0121; 0.0211) |
| Diarrhea | 0.0152 (0.0117; 0.0194) |
| Measles | 0.0023 (0.0008; 0.005) |
| Hepatitis | 0.0009 (0.0001; 0.0026) |
| Other infections | 0.0915 (0.0818; 0.102) |
| *All infections* | 0.3962 (0.3793; 0.4133) |

*Calculated from the subset of 710 (26.3%) patients with registered infections, total person-years at risk: 5,181
