# Supplementary material for: The burden of common variable immunodeficiency disorders: a retrospective analysis of the European Society for Immunodeficiency (ESID) registry data
Source: Orphanet J Rare Dis. 2018 Nov 12;13:201. doi: 10.1186/s13023-018-0941-0 (PMC6233554; doi:10.1186/s13023-018-0941-0)
Supplement: Supplementary file 6 — Burden of disease to society and the individual patient. (DOCX 15 kb) [file 13023_2018_941_MOESM6_ESM.docx]

Additional file 6. Burden of disease to society and the individual patient

Table A. Burden of disease to society: annual age-standardized DALY rate associated with CVID and top-ten health problems in Europe, from high to low

| Health problem | Burden to society: DALY (95%CI) per 100,000 general population¹ |
| --- | --- |
| Back and neck pain | 1,712 (1,203; 2,218) |
| Ischemic heart disease | 1,125 (1,076; 1,184) |
| Depressive disorders | 644 (426; 874) |
| Tracheal, bronchus, and lung cancer | 635 (617; 658) |
| Stroke | 543 (505; 594) |
| Alzheimer disease and other dementias | 454 (389; 535) |
| Diabetes mellitus | 389 (319; 469) |
| Chronic obstructive pulmonary disease | 356 (338; 375) |
| Colon and rectum cancer | 310 (296; 328) |
| Lower respiratory infections | 187 (176; 200) |
| **CVID** | **1.5 (1.3; 1.7)** |

¹ Source: Global Burden of Disease Studies, Western Europe, 2015: <http://ghdx.healthdata.org/gbd-results-tool>

Table B. Burden of disease to individual patient: annual age-standardized DALY rate associated with CVID and top-ten health problems in Europe, from high to low

| Health problem | Burden to patient: DALY (95%CI) per 100,000 diagnosed population² |
| --- | --- |
| Tracheal, bronchus, and lung cancer | 1,096,432 (1,089,525; 1,115,284) |
| Lower respiratory infections | 397,566 (396,919; 402,394) |
| Colon and rectum cancer | 183,419 (180,282; 189,104) |
| Alzheimer disease and other dementias | 66,898 (65,993; 69,658) |
| Stroke | 60,247 (59,032; 62,952) |
| Ischemic heart disease | 52,953 (52,911; 53,339) |
| **CVID** | **36,785 (33,078; 41,380)** |
| Depressive disorders | 16,710 (11,756; 21,054) |
| Back and neck pain | 12,057 (8,960; 14,778) |
| Diabetes mellitus | 12,043 (10,591; 13,620) |
| Chronic obstructive pulmonary disease | 10,445 (10,287; 10,592) |

² Calculated as DALY per 100,000 general population X 100,000/ disease prevalence per 100,000 general population
