# Supplementary material for: The burden of common variable immunodeficiency disorders: a retrospective analysis of the European Society for Immunodeficiency (ESID) registry data
Source: Orphanet J Rare Dis. 2018 Nov 12;13:201. doi: 10.1186/s13023-018-0941-0 (PMC6233554; doi:10.1186/s13023-018-0941-0)
Supplement: Supplementary file 7 — All-cause mortality table, CVID cohort. (DOCX 16 kb) [file 13023_2018_941_MOESM7_ESM.docx]

Additional file 7. All-cause mortality table, CVID cohort

| **Time since diagnosis (years)** | **Survival (%)** | **Lower limit 95% CI** | **Upper limit 95% CI** |
| --- | --- | --- | --- |
| 1 | 1.00 | 1.00 | 1.00 |
| 2 | 1.00 | 0.99 | 1.00 |
| 3 | 0.99 | 0.99 | 1.00 |
| 4 | 0.99 | 0.98 | 1.00 |
| 5 | 0.99 | 0.98 | 0.99 |
| 6 | 0.98 | 0.97 | 0.99 |
| 7 | 0.98 | 0.97 | 0.99 |
| 8 | 0.97 | 0.95 | 0.98 |
| 9 | 0.95 | 0.94 | 0.97 |
| 10 | 0.95 | 0.93 | 0.97 |
| 11 | 0.94 | 0.92 | 0.96 |
| 12 | 0.93 | 0.91 | 0.95 |
| 13 | 0.91 | 0.89 | 0.94 |
| 14 | 0.90 | 0.87 | 0.92 |
| 15 | 0.89 | 0.86 | 0.92 |
| 16 | 0.89 | 0.86 | 0.92 |
| 17 | 0.86 | 0.83 | 0.90 |
| 18 | 0.84 | 0.80 | 0.88 |
| 19 | 0.82 | 0.78 | 0.86 |
| 20 | 0.81 | 0.77 | 0.86 |
| 21 | 0.81 | 0.77 | 0.85 |
| 22 | 0.79 | 0.75 | 0.84 |
| 23 | 0.77 | 0.72 | 0.82 |
| 25 | 0.76 | 0.71 | 0.81 |
| 26 | 0.73 | 0.67 | 0.79 |
| 27 | 0.72 | 0.66 | 0.78 |
| 28 | 0.71 | 0.65 | 0.77 |
| 29 | 0.68 | 0.61 | 0.75 |
| 34 | 0.63 | 0.55 | 0.72 |
| 35 | 0.61 | 0.52 | 0.70 |
| 38 | 0.58 | 0.49 | 0.69 |
| 39 | 0.55 | 0.44 | 0.67 |
| 45 | 0.49 | 0.37 | 0.66 |
| 49 | 0.35 | 0.17 | 0.72 |

*based on 2,700 patient records
