# Supplementary material for: The burden of common variable immunodeficiency disorders: a retrospective analysis of the European Society for Immunodeficiency (ESID) registry data
Source: Orphanet J Rare Dis. 2018 Nov 12;13:201. doi: 10.1186/s13023-018-0941-0 (PMC6233554; doi:10.1186/s13023-018-0941-0)
Supplement: Supplementary file 8 — Diagnostic criteria of CVID. (DOCX 15 kb) [file 13023_2018_941_MOESM8_ESM.docx]

Additional file 8. Diagnostic criteria of CVID

In 2014, the ESID registry published the following working definitions for clinical diagnosis of CVID (7):

At least one of the following:

- increased susceptibility to infection/ autoimmune manifestations/ granulomatous disease/ unexplained polyclonal lymphoproliferation/ affected family member with antibody deficiency

AND marked decrease of IgG and marked decrease of IgA with or without low IgM levels (measured at least twice; <2SD of the normal levels for their age);

AND at least one of the following:

- poor antibody response to vaccines (and/ or absent isohaemagglutinins); i.e. absence of protective levels despite vaccination/ low switched memory B-cells (<70% of age-related normal value)

AND secondary causes of hypogammaglobulinemia have been excluded

AND diagnosis is established after the 4th year of life (but symptoms may be present before)

AND no evidence of profound T-cell deficiency, defined as 2 out of the following (y=years of life):

- CD4 numbers/microliter: 2-6y <300, 6-12y <250, >12y <200/ % naive CD4 : 2-6y <25%, 6-16y <20%, >16y <10%/ T cell proliferation absent.

In 2016, an international consensus “ICON” was published suggesting less stringent diagnostic criteria (3):

At least one of the characteristic clinical manifestations (infection, autoimmunity, lymphoproliferation),

OR asymptomatic individuals who fulfil the following criteria:

1. A repeatedly marked decrease in IgG levels (>2 SDs less than the mean for age).
2. A marked decrease in levels of at least 1 of the isotypes IgM or IgA
3. diagnosis of immunodeficiency at greater than 4 years of age;
4. Absent isohemagglutinins, poor response to vaccines, or both; and
5. Exclusion of defined causes of hypogammaglobulinemia.
